# Antifungal Activity and Multi-Target Mechanism of Action of Methylaervine on *Candida albicans*

**DOI:** 10.3390/molecules29184303

**Published:** 2024-09-11

**Authors:** Jinyi Liu, Luyao Wang, Yifan Sun, Yingyan Xiong, Runchu Li, Meixia Sui, Zhenzhen Gao, Wei Wang, Hao Sun, Jiangkun Dai

**Affiliations:** 1 School of Life Science and Technology, Shandong Second Medical University, Weifang 261053, China; 18385287713@163.com (J.L.); wangluyao_lzy@163.com (L.W.); 17861203076@163.com (Y.S.); xiongyingyan@outlook.com (Y.X.); 13869636860@139.com (R.L.); 2 College of Biology and Oceanography, Weifang University, Weifang 261061, China; 3 School of Pharmaceutical Sciences, Liaocheng University, Liaocheng 252059, China; gaozhenzhen@lcu.edu.cn; 4 School of Pharmacy, Shandong Second Medical University, Weifang 261053, China; wangw55@sdsmu.edu.cn

**Keywords:** methylaervine, antifungal, *Candida albicans*, multi-target mechanism of action

## Abstract

The discovery of a lead compound against *Candida albicans* is urgently needed because of the lack of clinically available antifungal drugs and the increase in drug resistance. Herein, a *β*-carboline alkaloid methylaervine (MET) exhibited potential activity against *C. albicans* (MIC = 16–128 μg/mL), no hemolytic toxicity, and a low tendency to induce drug resistance. An antifungal mechanism study indicated that MET effectively inhibited the biofilm formation and disrupted the mature biofilm. Moreover, filamentation formation and spore germination were also weakened. The electron microscopy analysis revealed that MET could damage the cell structure, including the cell wall, membrane, and cytoplasm. In particular, the permeability and integrity of the cell membrane were destroyed. When it entered the fungi cell, it interfered with the redox homeostasis and DNA function. Overall, MET can inhibit the growth of *C. albicans* from multiple channels, such as biofilm, filamentation, cell structure, and intracellular targets, which are difficult to mutate at the same time to generate drug resistance. This work provides a promising lead compound for the creation of new antifungal agents against *C. albicans*.

## 1. Introduction

Invasive fungal infections caused by *Candida albicans* seriously threaten human health, especially for the immunocompromised hosts, such as surgical and cancer chemotherapy patients [1,2]. Although opportunistic, the fungi can infect mammals ranging from superficial skin lesions to deep organs, causing oral thrush, vaginitis, and bloodstream and deep organ infections [3]. Currently, clinically available antifungal drugs against *C. albicans* mainly include azoles, polyenes, and echinocandins, which not only have inherent flaws, such as hepatorenal toxicity and limited efficacy, but also lead to the development of resistance because they only target essential fungal process sites, such as the fungal membrane or fungal wall [4]. Therefore, new antifungal leads against *C. albicans* with new therapeutic approaches over conventional therapies need further discovery.

Clinical studies have found that single-target drugs easily produce gene mutations and can result in drug resistance, while single-target drug combinations or multi-target drugs can act on diverse targets in the whole cell at the same time, which is difficult to mutate in the meantime and has synergistic effects [5]. In particular, multi-target drugs have superior therapeutic efficacy, lower risk of drug interaction and side effects, and more predictable pharmacokinetic and pharmacodynamic characteristics, and they are the best strategy to prevent the generation and development of drug resistance [6].

**Figure 1:**
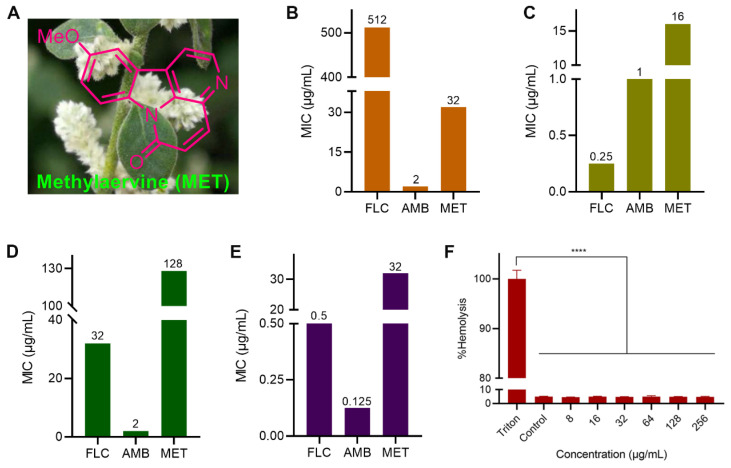
In vitro antifungal and hemolytic activities of MET. (**A**) Chemical structure of MET; (**B**) antifungal susceptibility of *C. albicans* ATCC 90028; (**C**) antifungal susceptibility of *C. albicans* ATCC 90029; (**D**) antifungal susceptibility of *C. albicans* ATCC 10231; (**E**) antifungal susceptibility of *C. albicans* ATCC 14053; (**F**) hemolytic activity of MET against sheep red blood cells (RBCs), **** *p* < 0.0001 vs. the positive control Triton X-100 (0.1%).

Natural products have been an important source for the discovery of original antimicrobial agents because of their unique chemical structure, diverse targets, and advantages in avoiding cross-resistance [7]. *β*-Carboline alkaloids have attracted extensive attention recently with nine successfully marketed drugs, which also exhibit potential antifungal activity [8]. Methylaervine (MET), attributed to *β*-carboline alkaloids, being first isolated from the herb *Aerva lanata* Juss. in 1991 (Figure 1A), has displayed many kinds of pharmacological activities, such as antitumor, antibacterial, antiviral, and antifungal effects [9,10,11,12,13]. Previously, the antifungal potential of MET against agricultural pathogenic fungi *Alternaria solani*, *Fusarium graminearum*, and *Fusarium solani* was assessed by our group [11]. However, its activity and mechanism against *C. albicans* were not clear still. The objective of this work is to demonstrate the potential of MET as a promising lead compound against *C. albicans* and explore its multi-target mechanism of action.

## 2. Results and Discussion

### 2.1. Antifungal Activity against C. albicans

Candida species are known for their remarkable adaptability, diverse virulence factors, and complex pathogenic mechanisms. Each *C. albicans* isolate exhibits unique virulence and phenotypic characteristics, making them intriguing subjects for study. Despite their genetic variances, these strains have not evolved into distinct species. This makes it crucial to consider the potential for different inhibitory activities of the same compound against various strains of *C. albicans* [1,3]. The strains of *C. albicans* ATCC 90028, ATCC 90029, and ATCC 14053 are derived from blood. The *C. albicans* ATCC 10231 is derived from a patient with bronchomycosis. Among them, the *C. albicans* ATCC 90029 is attributed to drug-resistant fungus. The antifungal activity of MET (Figure 1A) and positive drugs fluconazole (FLC) and amphotericin B (AMB) against *C. albicans* were evaluated by microdilution assays [14]. As shown in Figure 1B, MET inhibited the growth of *C. albicans* ATCC 90028 with a minimum inhibitory concentration (MIC) of 32 μg/mL, which was superior to the positive drug FLC. The MIC of MET against *C. albicans* ATCC 90029 was 16 μg/mL (Figure 1C). However, MET exhibited weak antifungal activity against *C. albicans* ATCC 10231 with MIC of 128 μg/mL (Figure 1D). The antifungal activity of MET against *C. albicans* ATCC 14053 was the same as that against ATCC 90028 (Figure 1E). In order to explore the antifungal activity and mechanism in depth, the sensitive strain *C. albicans* ATCC 90029 was selected for further study.

### 2.2. Hemolytic Activity

The hemolytic activity of antifungal agents is an important index in estimating biocompatibility [15,16]. A previous study showed that the antifungal drug AMB could result in approximately 80% hemolysis at 4 μg/mL [15]. When AMB’s concentration was greater than 8 μg/mL, the hemolysis rate reached 100%. In this study (Figure 1F), no hemolysis was evident even when the concentrations of MET reached 16× MIC (256 μg/mL). The results revealed that MET was biocompatible for therapeutic application.

### 2.3. Growth Inhibition Activity

**Figure 2:**
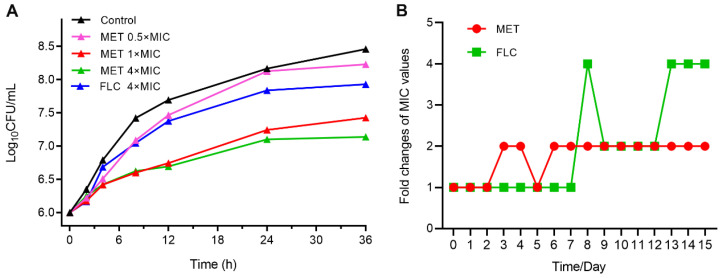
Growth kinetics and drug-resistance evaluation. (**A**) The time–growth curves of MET against *C. albicans* ATCC 90029; (**B**) resistance study against *C. albicans* ATCC 90029 with 15 passages of repeated exposure to 0.5× MIC of MET.

For the further evaluation of the antifungal efficacy of MET, time–growth curves were created [17]. As shown in Figure 2A, MET inhibited the proliferation of *C. albicans* ATCC 90029 cells with a concentration-dependent effect. Compared to the control group, a 1.03 log reduction of the viable count of *C. albicans* ATCC 90029 was displayed after treated by 1× MIC of MET for 36 h. Overall, MET could inhibit the growth of *C. albicans* ATCC 90029 under effective doses.

### 2.4. Resistance Development Evaluation

The developing resistance is a major challenge for the treatment of infectious disease [18]. The positive drug FLC was used as a control in the assay. The propensity of *C. albicans* ATCC 90029 to develop drug resistance was evaluated by repeated exposure to MET (0.5× MIC) with 15 passages. As depicted in Figure 2B, the fold changes of MIC increased to 4-fold and 2-fold for FLC and MET, respectively. The results demonstrated that MET had a low tendency to induce fungal resistance during 15 passages.

### 2.5. Biofilm Formation Inhibition and Eradication Activity

**Figure 3:**
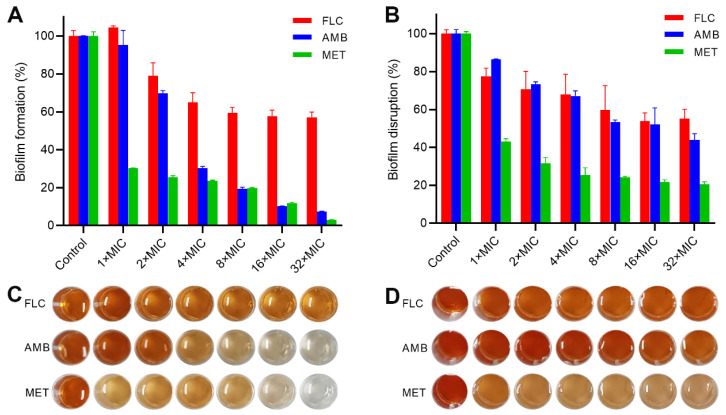
Effect of MET on biofilms. (**A**) Biofilm formation inhibition of *C. albicans* ATCC 90029; (**B**) disruption of mature biofilms of *C. albicans* ATCC 90029; (**C**) XTT staining images for biofilm formation assay; (**D**) XTT staining images for biofilm disruption assay.

The fungal biofilm is highly structured by self-produced extracellular matrixes, such as protein, exopolysaccharide, lipids, and nucleic acids, which provide a protective barrier for the fungi cells [14]. In particular, the ability to form biofilms is one of the cores for pathogenesis, which is also an important factor in the development of drug resistance of *C. albicans* [19,20]. A large body of research indicated that *C. albicans* biofilms are inherently resistant to the currently available conventional antifungal agents. Therefore, it is crucial to discover effective antifungal molecules against biofilms. The 2,3-bis(2-methoxy-4-nitro-5-sulfophenyl)-2*H*-tetrazolium-5-carboxanilide (XTT) reduction assay was examined to determine the effect of MET on biofilm formation and the disruption of preformed biofilms [19]. As shown in Figure 3A,C, MET and AMB effectively inhibited biofilm formation in a dose-dependent mode. Approximately 70% of biofilm inhibition of MET was achieved at 1× MIC. By contrast, only 43% of biofilm inhibition of FLC was obtained at 32× MIC. Thus, MET has the potential to inhibit biofilm formation. Notably, the result may be associated with the growth inhibition caused by MET. As shown in Figure 3B,D, MET could eradicate 57% of preformed biofilms at 1× MIC (16 μg/mL). In contrast, only 48% of preformed biofilms were disrupted by the positive drug AMB (16 μg/mL). Compared to the drug-free control, MET dose-dependently destructed the mature biofilms. Ultimately, ~80% of the mature biofilm was eradicated by 32× MIC of MET. These results indicated that MET could inhibit the biofilm formation and disrupt the preformed mature biofilms.

### 2.6. Effects on Filamentation

**Figure 4:**
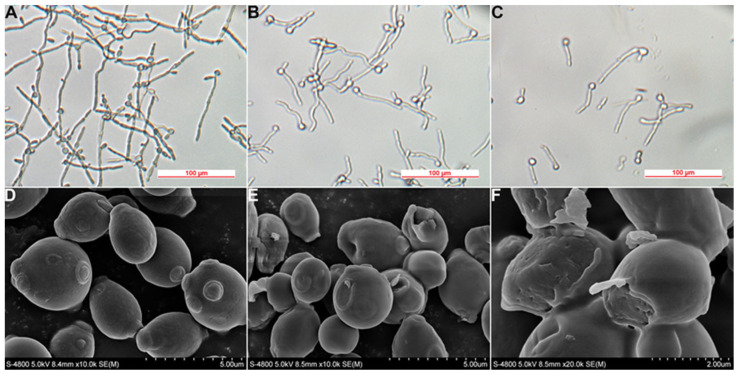
Alterations of filamentation and cell surface of *C. albicans* ATCC 90029. (**A**) The inhibition effects on the filamentation (0× MIC, control group); (**B**) the inhibition effects on the filamentation (1× MIC); (**C**) the inhibition effects on the filamentation (4× MIC); (**D**) an SEM image of *C. albicans* with the scale bar of 5 µm (0× MIC); (**E**) an SEM image of *C. albicans* with a scale bar of 5 µm (1× MIC); (**F**) an SEM image of *C. albicans* with a scale bar of 2 µm (1× MIC).

Hyphal formation as a virulence factor of *C. albicans* plays a key role in pathogenicity, which can easily invade mucosal tissue and cause invasive fungal infections [20]. Moreover, hyphae of *C. albicans* can facilitate the escape from immune surveillance and the formation of fungal biofilms [17]. Therefore, the effects of MET for inhibiting *C. albicans* filamentation were studied. As shown in Figure 4A, the filamentous structures were dense and disorganized with long hyphae. Interestingly, the hyphal growth of *C. albicans* was inhibited gradually with increasing concentrations of MET (Figure 4B,C). It was noticed that 4× MIC of MET significantly inhibited the formation of filaments, with the appearance of yeast type (Figure 4C).

### 2.7. Effects on Morphology of Fungal Spore

Spores are reproductive structures of fungi, which are required for pathogenesis and long-term survival for *C. albicans*. Spore germination, as the key process to initiate vegetative growth, has been used as a specific target for inhibiting fungus growth [21]. Therefore, we investigated the morphological changes of spores by scanning electron microscopy (SEM). For the control group, the cell surface was plump and intact, with clear and distinct spore germination sites (Figure 4D). After being treated with MET, the fungi cells became wrinkled, accompanied by a concave depression. The germination sites were not obvious or even slightly concave (Figure 4E). Moreover, obvious fungi cell structure damage can also be observed in Figure 4F, such as on the cell wall and membrane. The above results also confirmed the growth and filamentation inhibition effects of MET on *C. albicans*.

### 2.8. Effects on Ultrastructural Features of Spore

**Figure 5:**
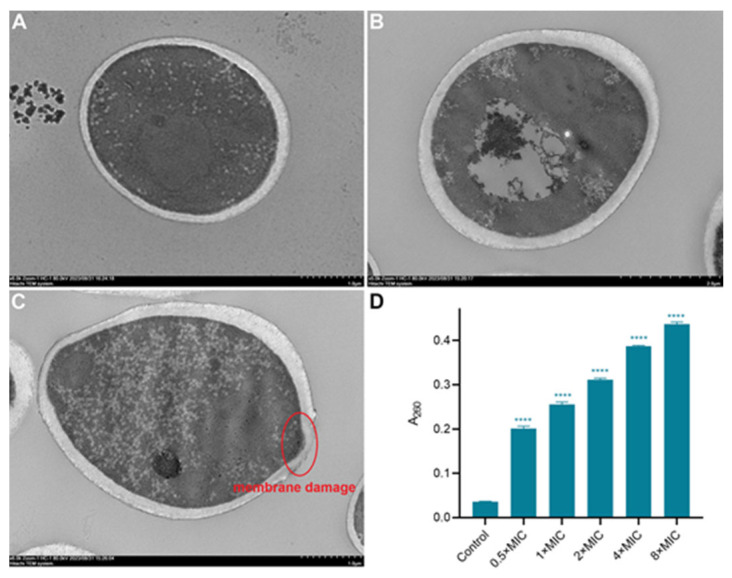
Alterations of the internal microstructure of *C. albicans* ATCC 90029. (**A**) A TEM image of normal *C. albicans* with a scale bar of 1 µm; (**B**) a TEM image of *C. albicans* treated by 1× MIC of MET with a scale bar of 2 µm; (**C**) a TEM image of *C. albicans* treated by 1× MIC of MET with a scale bar of 1 µm; (**D**) the level of nucleic acid leakage under different concentrations of MET, **** *p* < 0.0001 vs. control group.

The cell wall and cell membrane of *C. albicans* are important for cell viability and morphology and can cause fungi cell death with slight alterations to the structural integrity [22,23]. Herein, we determined the alteration of the internal microstructure of *C. albicans* by using a transmission electron microscope (TEM) (Hitachi, Tokyo, Japan). In the control group, *C. albicans* cells displayed a well-defined cell wall and membrane with clear cell boundaries and normal electron density in the cytoplasm (Figure 5A). After being treated with 1× MIC of MET, the fungi cells’ shape became irregular, and the thickness of the cell wall was thicker and uneven (Figure 5B,C). Moreover, the abnormal electron density was observed in the cytoplasm. The damaged area of the plasma membrane is shown in Figure 5C, which would result in the outflow of intracellular lysate.

### 2.9. Effects on Membrane Permeability

For the investigation of the influence of MET on cell membrane permeability, nucleotides outside the *C. albicans* were determined by the intensity of absorption at 260 nm [24]. As shown in Figure 5D, the level of nucleic acid released significantly increased with increasing concentrations of MET, indicating that the cell membrane permeability was changed.

### 2.10. Effects on Membrane Integrity

**Figure 6:**
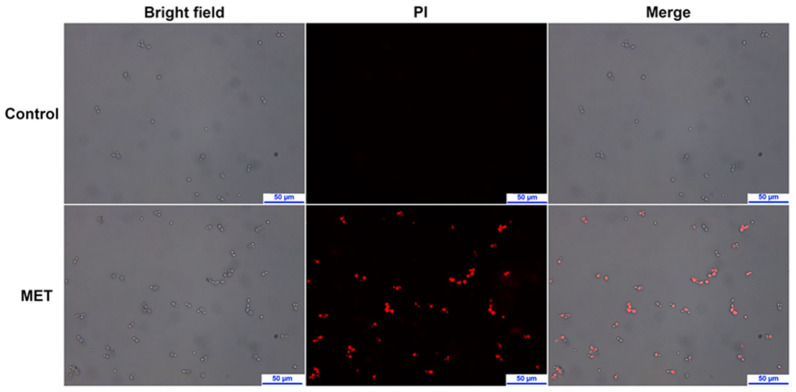
The effect on membrane integrity after treated by 1× MIC of MET, visualized with a fluorescence microscope using bright field and PI staining.

Fluorescent dye propidium iodide (PI), as a cell membrane impermeable agent, can only enter the fungi cells when the integrity of the membrane is damaged and emits red fluorescence [25]. As depicted in Figure 6, the *C. albicans* cells were observed in the bright field, and no red fluorescence was detected in the control group. However, *C. albicans* cells exposed to MET showed significant red fluorescence. The results revealed that MET could damage the integrity of *C. albicans* cell membrane.

### 2.11. Effects on Reactive Oxygen Species (ROS) Level

**Figure 7:**
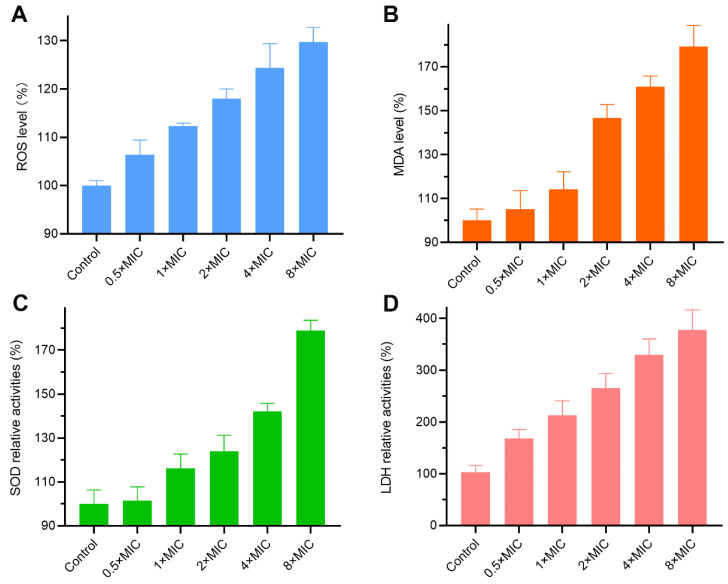
Intracellular changes of *C. albicans* ATCC 90029 after treatment with different concentrations of MET. (**A**) Effect on intracellular ROS accumulation; (**B**) effect on intracellular MDA levels; (**C**) effect on the activities of SOD; (**D**) effect on the activities of LDH.

The intracellular ROS induction and accumulation can cause oxidative damage to biomacromolecules, such as lipids, DNA, and proteins, ultimately resulting in cell death [25]. As depicted in Figure 7A, ROS levels in *C. albicans* increased significantly after treatment with MET for 6 h. Compared with the control group, the ROS levels increased by 1.12- and 1.29-fold for 1× and 8× MIC, respectively. The results revealed that MET could result in ROS overproduction in *C. albicans*.

### 2.12. Effects on Malondialdehyde (MDA) Level

As is known, the intracellular accumulation of ROS can lead to membrane lipid peroxides accompanied by MDA generation [26]. The MDA level was also measured to investigate the oxidative stress effects. As depicted in Figure 7B, the amount of generated MDA increased gradually in a dose-dependent manner. Specifically, the increment reached 1.14- and 1.79-fold when *C. albicans* cells were treated with 1× and 8× MIC of MET, respectively.

### 2.13. Effects on Activity of Superoxide Dismutase (SOD)

SOD, as an important antioxidant enzyme in *C. albicans*, can be involved in the detoxification of superoxide radicals, which closely relates to sudden oxidative damage defense [27]. As shown in Figure 7C, MET treatment led to an increase in SOD activity in a dose-dependent manner. The intracellular mean SOD activities increased 1.16- and 1.78-fold with 1× and 8× MIC of MET, respectively. The above results indicated that redox homeostasis of *C. albicans* was intervened by MET.

### 2.14. Effects on Activity on Energy Production

LDH is one of the important mitochondrial dehydrogenases in the biosynthesis of ATP that can catalyze the conversion of lactate to pyruvate in the anaerobic glycolysis pathway [28]. For the exploration of the effect of MET on energy production in fungi cells, the LDH activities were determined. As depicted in Figure 7D, MET exhibited a dose-dependent activation effect on the activities of intracellular LDH, which might be a stress response to an adverse environment. Succinate dehydrogenase (SDH) plays an important role in the tricarboxylic acid (TCA) cycle, which is a central metabolic process in mitochondria that produces energy. In our previous study, we found that MET could inhibit the activity of SDH by using docking simulation [11]. The above results indicated that the energy system of *C. albicans* was influenced by MET.

### 2.15. Transcriptome Analysis

**Figure 8:**
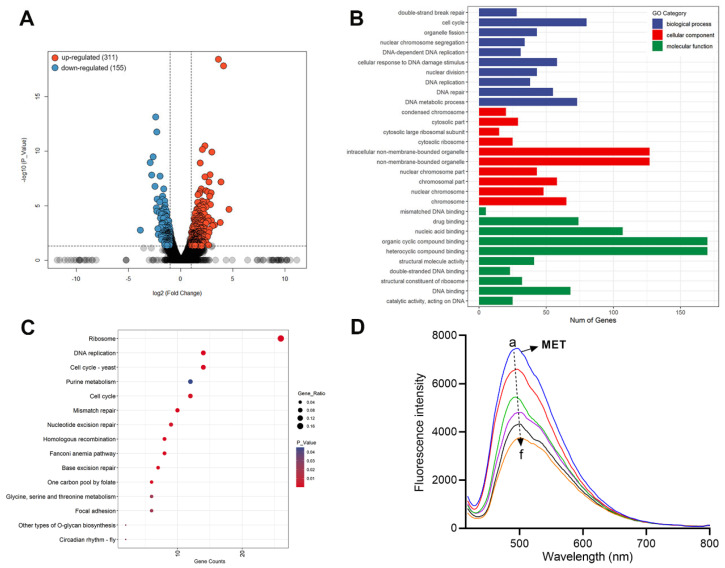
Transcriptome analysis and fluorescence spectra titration. (**A**) A volcano plot showing the DEGs; (**B**) the top enriched GO terms; (**C**) the enriched KEGG pathways; (**D**) fluorescence spectra of MET (5 × 10^−5^ M) in the presence of indicated concentrations of ct-DNA (0–2.5 × 10^−5^ M).

Transcriptome sequencing analysis was further carried out to comprehensively explore the antifungal mechanism [26,29]. A total of 6263 expressed genes were changed for *C. albicans* cells after treatment with 16 μg/mL of MET for 6 h. As depicted in Figure 8A, 466 differentially expressed genes (DEGs) met the cut-off criteria (*p*-value < 0.05 and |log_2_FC| > 1), including 311 up-regulated and 155 down-regulated genes. Furthermore, we performed gene ontology (GO) functional annotation and Kyoto Encyclopedia of Genes and Genomes (KEGG) enrichment analysis to systematically investigate the biological functions and pathways. As displayed in Figure 8B, the representative Go terms were presented, including biological processes, molecular functions, and cellular components. For the biological processes, double-strand break repair (*P* = 1.7 × 10^−11^), nuclear chromosome segregation (*P* = 2.2 × 10^−12^), DNA-dependent DNA replication (*P* = 1.9 × 10^−12^), cellular response to DNA damage stimulus (*P* = 5.2 × 10^−13^), DNA replication (*P* = 1.7 × 10^−13^), DNA repair (*P* = 1.1 × 10^−13^), and the DNA metabolic process (*P* = 3.7 × 10^−15^) closely related to the DNA target were significantly disturbed [30], indicating that MET may directly target to DNA. Moreover, the cell cycle (*P* = 8.3 × 10^−12^) and nuclear division (*P* = 5.1 × 10^−13^) related to the reproduction of spores were also intervened [31]. For cellular components, the chromosome, ribosome, and cytosolic part were mainly changed, which conformed to the results of TEM, indicating that MET may damage the structure and function of the subcellular organelle of *C. albicans*. For the molecular functions, MET influenced the functions of mismatched DNA binding (*P* = 0.0006), drug binding (*P* = 0.0005), DNA binding (*P* = 1.7 × 10^−6^), nucleic acid binding (*P* = 0.0001), organic cyclic compound binding (*P* = 9.6 × 10^−5^), heterocyclic compound binding (*P* = 7.3 × 10^−5^), double-stranded DNA binding (*P* = 3.4 × 10^−5^), and so on, which also indicated that MET could bind to DNA [32]. Based on KEGG pathway analysis, 173 pathways were enriched, and the top 15 are displayed in Figure 8C. Among these, DNA replication (*P* = 1.7 × 10^−7^), mismatch repair (*P* = 1.5 × 10^−6^), homologous recombination (*P* = 6.4 × 10^−6^), and nucleotide excision repair (*P* = 0.0027) were significantly disturbed, which closely related to DNA function [30,31,32]. In addition, pathways associated with spore reproduction, such as cell cycle (*P* = 0.0001); purine metabolism (*P* = 0.04); glycine, serine, and threonine metabolism (*P* = 0.02); and focal adhesion (*P* = 0.02), were also influenced. Transcriptome sequencing analysis mainly revealed that MET could bind to DNA and destroy its function.

### 2.16. Interaction with DNA

In order to further investigate the interaction between MET and DNA, fluorescence spectroscopy titration was performed using calf thymus DNA (ct-DNA) as the model [33,34]. As shown in Figure 8D, the fluorescence intensity of MET at 494 nm decreased with the proportional addition of ct-DNA. Moreover, slight red-shift was observed. The results revealed that MET could bind with DNA to block the growth and reproduction of *C. albicans* cells.

### 2.17. In Silico Prediction of Toxicity

Considering the DNA binding capacity of MET, the toxicity and mutagenicity were further evaluated using a webserver, ProTox 3.0 [35]. As shown in Table 1, the median lethal dose (LD_50_) was 450 mg/Kg, much higher than the effective drug dose (16 μg/mL). Moreover, it also exhibited no organ toxicity against the liver, kidney, respiratory system, and heart. In this prediction, however, the compound MET was likely to be active in mutagenicity, which should be attenuated in future drug design.

## 3. Materials and Methods

### 3.1. Reagents and Strains

MET (CAS: 86293-40-5) was synthesized by our group previously [11]. The positive drugs FLC and AMB were provided by Macklin (Shanghai, China). The ROS, MDA, SOD, and LDH assay kits were obtained from Solarbio (Beijing, China). Dimethyl sulfoxide (DMSO), PI, XTT, and triton X-100 were purchased from Beyotime (Shanghai, China). The Roswell Park Memorial Institute (RPMI 1640) medium was purchased from Thermo Fisher Scientific (Waltham, MA, USA). The strains of *C. albicans* (ATCC 90028, ATCC 90029, ATCC 10231, and ATCC 14053) were purchased from China General Microbiological Culture Collection Center.

### 3.2. In Vitro Antifungal Activity Assay

According to the previous method [14], the in vitro antifungal activity is expressed by minimum inhibitory concentration (MIC) values, which inhibits 80% of yeast growth. The MIC values for each compound were evaluated using the serial dilution method. Strains were generally maintained in glycerol (20%) at −20 °C. After incubating in yeast extract peptone dextrose (YEPD) medium (35 °C, 220 rpm) for 24 h, they were inoculated to Sabouraud Dextrose Agar (SDA) plates and cultured at 35 °C for 48 h. Then, the fungal cell was cultured to the exponential growth phase in YEPD medium at 35 °C. The *C. albicans* cells were harvested and finally diluted to 1 × 10^3^ CFU/mL with RPMI 1640 medium. The concentration of tested compounds (MET, FLC, and AMB) ranged from 0.0625 to 512 μg/mL. A blank control and negative control were also set. After incubation at 35 °C for 24 h, the optical density at 630 nm (OD_630_) of each well of the 96-well plates was determined using a BioTek ELX-800 microplate reader (BioTek, Winooski, VT, USA). After subtracting the background, the MIC values were calculated.

### 3.3. Hemolytic Activity Assay

According to the previous method [15,16], fresh RBCs were obtained from sheep blood. Then, the RBCs suspension (4% *v*/*v*, 250 μL) was added to the MET (250 μL) solutions with different concentrations in 2 mL via a centrifuge tube. After incubation (37 °C, 1 h) and centrifugation (3000 rpm, 3 min), the supernate (100 μ*L*) was transferred to a 96-well plate. Triton X-100 (0.1%), DMSO solution, and phosphate-buffered saline (PBS) were used as the positive, negative, and blank controls, respectively. The absorbance values at 540 nm were obtained. Hemolysis (%) was represented as follows: $Hemolysis\left(\%\right)=\left[\left(A_{sample}-A_{blank\;control}\right)÷\left(A_{positive\;control}-A_{blank\;control}\right)\right]\times 100$.

### 3.4. Time−Growth Curve Assay

According to the previous method [17], the exponentially growing *C. albicans* ATCC 90029 were harvested, washed with PBS, and resuspended in RPMI 1640 medium to a concentration of 1 × 10^6^ CFU/mL. The indicated concentrations of MET (0×, 0.5×, 1×, and 4× MIC) and FLC (4× MIC) were added and cultured at 35 °C (220 rpm) for 36 h. At the indicated time points (0, 2, 4, 8, 12, 24, and 36 h), the fungal cells were counted using a cell-counting plate.

### 3.5. Drug Resistance Assay

According to the previous method [19], the developing resistances of MET and FLC were explored by serial passage microdilution assays. The *C. albicans* ATCC 90029 suspension in RPMI 1640 medium was incubated with MET or FLC at a concentration of 0.5× MIC (35 °C, 220 rpm) for 24 h to make new *C. albicans*. After the new fungal cells were cultured to the exponential growth phase in YEPD medium at 35 °C for 16 h, the new MIC values for new *C. albicans* were assessed. The entire process was repeated for 15 passages.

### 3.6. Biofilm Formation Inhibition Assay

According to the previous method [14,19], 100 μL of the exponentially growing *C. albicans* ATCC 90029 (2 × 10^6^ CFU/mL) was added into a 96-well plate. Then, 100 μL volumes of MET, FLC, or AMB with final concentrations of 1×, 2×, 4×, 8×, 16×, and 32× MIC were inoculated into the 96-well plate. The *C. albicans* ATCC 90029 suspension was used as the control. After being cultured at 37 °C for 24 h, the medium was aspirated, and each well was carefully washed twice with PBS. The biofilm formation was semi-quantitatively determined using an XTT reduction assay. The OD_490_ of each well was determined using the BioTek ELX-800 microplate reader (Winooski, VT, USA).

### 3.7. Biofilm Disruption Assay

According to the previous method [14,19], 200 μL of the exponentially growing *C. albicans* ATCC 90029 (1 × 10^6^ CFU/mL) was added into a 96-well plate and cultured at 37 °C for 24 h. After removing the medium and phytoplankton cells, the mature biofilm was carefully washed with PBS. Then, 200 μL volumes of MET, FLC, or AMB with final concentrations of 0×, 1×, 2×, 4×, 8×, 16× and 32× MIC were inoculated into the 96-well plate. The 96-well plate was incubated at 37 °C for another 24 h. After discarding the medium and carefully washing the samples with PBS, the disruption of the performed biofilm was also determined using the XTT reduction assay.

### 3.8. In Vitro Hyphal Formation Assay

According to the previous method [17,35], 1.5 mL of the exponentially growing *C. albicans* ATCC 90029 (5 × 10^5^ CFU/mL) was added to a 12-well plate. The indicated concentrations (0×, 1×, and 4× MIC) of MET were added, and then, the plates were cultured at 37 °C for 4 h. Finally, the morphology of *C. albicans* ATCC 90029 cells was recorded using the Nikon TS2-FL inverted microscope (Nikon, Tokyo, Japan).

### 3.9. SEM Assay

According to the previous method [7,36], 10 mL of the exponentially growing *C. albicans* ATCC 90029 (1 × 10^6^ CFU/mL) in YEPD medium was added to the 15 mL sterile centrifuge tube. For the treated group, the fungi cells were cultured with 1× MIC of MET (35 °C, 220 rpm) for 4 h. For the control group, a corresponding DMSO solution was added. After removing the medium and washing with PBS, the *C. albicans* ATCC 90029 were harvested and then fixed with 2.5% glutaraldehyde. After dehydration with different concentrations of ethanol and drying with hexamethyldisilane, a gold-sputtering process was performed. Finally, the fungi cells were observed using a Hitachi S-4800 SEM (Hitachi, Tokyo, Japan) in a SCI-GO instrument test platform (https://www.sci-go.com/home, accessed on 4 September 2024).

### 3.10. TEM Assay

According to the previous method [37], 10 mL of the exponentially growing *C. albicans* ATCC 90029 (1 × 10^6^ CFU/mL) in YEPD medium was added to the 15 mL sterile centrifuge tube. For the treated group, the fungi cells were cultured with 1× MIC of MET (35 °C, 220 rpm) for 4 h. For the control group, a corresponding DMSO solution was added. After the fixation, dehydration, embedding, and slice processes, the fungi cells were observed using a Hitachi HC-1 TEM (Tokyo, Japan) in the SCI-GO instrument test platform (https://www.sci-go.com/home, accessed on 4 September 2024).

### 3.11. Nucleic Acid Leakage Assay

According to the previous method [24], the exponentially growing *C. albicans* ATCC 90029 was diluted to 1 × 10^6^ CFU/mL with PBS, and then, indicated concentrations of MET (0×, 0.5×, 1×, 2×, 4×, and 8× MIC) were added. After incubation at 35 °C for 4 h (220 rpm), the samples were centrifugated, and then OD_260_ values of the supernatant were measured by a UV-8000 ultraviolet spectrophotometer (YuanXi, Shanghai, China).

### 3.12. Live/Dead Cell Assay

According to the previous method [25], the exponentially growing *C. albicans* ATCC 90029 was diluted to 1 × 10^6^ CFU/mL, and then, indicated concentrations of MET (0× and 4× MIC) were added. After incubation at 35 °C for 4 h (220 rpm), the fungi cells were washed with PBS three times and resuspended in 200 μL of PBS. Then, 2 μL of PI (20 μg/mL) was added to the centrifuge tube, which was further cultured at 30 °C for 15 min in the dark. Finally, the fungi cells were visualized and analyzed using the Leica DM4 B microscope (Wetzlar, Germany).

### 3.13. Intracellular ROS Assay

According to the previous method [25], the exponentially growing *C. albicans* ATCC 90029 was diluted to 1 × 10^6^ CFU/mL, and then, indicated concentrations of MET (0×, 0.5×, 1×, 2×, 4×, and 8× MIC) were added. After incubation at 35 °C for 6 h (220 rpm), the fungi cells were washed with PBS three times and resuspended in 200 μL of DCFH-DA fluorescent dye. After treatment at 37 °C for 0.5 h in the dark, the fungi cells were harvested and washed twice with PBS. Then, the samples were resuspended with 1 mL of PBS. Finally, the fluorescence intensity with excitation and emission of 488 and 525 nm, respectively, was recorded using a Gemini EM microplate spectrofluorometer (Molecular Devices, San Jose, CA, USA).

### 3.14. Intracellular MDA Assay

According to the previous method [26], the exponentially growing *C. albicans* ATCC 90029 was diluted to 1 × 10^6^ CFU/mL, and then, indicated concentrations of MET (0×, 0.5×, 1×, 2×, 4×, and 8× MIC) were added. After incubation at 35 °C for 6 h (220 rpm), the fungi cells were collected and washed three times with PBS. Then, 0.1% of Triton X-100 solution (300 μL) was added, and the supernatant was obtained. The MDA relative content change was examined using MDA assay kits.

### 3.15. Intracellular SOD Assay

According to the previous method [27], the exponentially growing *C. albicans* ATCC 90029 was diluted to 1 × 10^6^ CFU/mL, and then, indicated concentrations of MET (0×, 0.5×, 1×, 2×, 4×, and 8× MIC) were added. After incubation at 35 °C for 6 h (220 rpm), the fungi cells were picked up and washed twice times with PBS. Then, the SOD relative activities were measured by SOD assay kits.

### 3.16. Intracellular LDH Assay

According to the previous method [28,38], the exponentially growing *C. albicans* ATCC 90029 was diluted to 1 × 10^6^ CFU/mL, and then, indicated concentrations of MET (0×, 0.5×, 1×, 2×, 4×, and 8× MIC) were added. After incubation at 35 °C for 6 h (220 rpm), the fungi cells were picked up and washed three times with PBS. Then, the supernatant was obtained by cell lysis and centrifugation. Finally, the LDH relative activities were measured by LDH assay kits.

### 3.17. RNA-seq Analysis

According to the previous method [26,29], the exponentially growing *C. albicans* ATCC 90029 was diluted to 1 × 10^6^ CFU/mL in the YEPD medium. Then, 50 mL of fungal suspension was added to the Erlenmeyer flask (150 mL). The fungi cells were cultured by 1× MIC of MET or corresponding DMSO at 35 °C for 6 h (220 rpm). Three biological replicates were performed. Then, the fungi cells were quickly harvested and washed with PBS. All groups were immediately frozen in liquid nitrogen for 10 min. Finally, the results were measured and analyzed by Shanghai Sangon Biotech Co., Ltd. (Shanghai, China).

### 3.18. Fluorescence Spectra Assay

According to the previous method [39], the fluorescence spectra (400–800 nm) were measured on a Hitachi F-4600 spectrophotometer (Japan) equipped with 1.0 cm quartz cells at room temperature. The stock solution of MET (5 × 10^−5^ M) was provided in Tris-HCl buffer solution (pH = 7.4). The ct-DNA ranging from 0 to 2.5 × 10^−5^ M was continuously added with incubation at 37 °C for 15 min. The excitation wavelength was set as 398 nm with an emission slit of 5 nm.

### 3.19. Toxicity Prediction Assay

The acute toxicity, organ toxicity, and mutagenicity of MET were predicted in the ProTox 3.0 web server, which is free and open for all users (https://tox.charite.de/protox3/, accessed on 4 September 2024) [35]. The TOX-PREDICTION module was selected, and the structure of MET was drawn in the ChemDoodle interface. Then, the data were obtained after clicking the button “Start Tox-Prediction”.

### 3.20. Statistical Analysis

All these assays were carried out in triplicate, and data are presented as mean ± standard deviation (SD). The significance of the difference was analyzed by Student’s *t*-test or one-way analysis of variance (ANOVA) with GraphPad Prism 8.0 software (CA, USA).

## 4. Conclusions

On account of the inherent side effects and accelerated evolution of drug-resistant mutants for clinical antifungal drugs, the new antifungal lead against *C. albicans* with the potential to prevent the development of drug resistance is urgently needed. Our current study reveals that MET has a potential antifungal effect against *C. albicans* with a peak MIC of 16 μg/mL, no hemolysis, and a low tendency to induce fungal resistance, confirming its feasibility as a lead framework for the design of antifungal drugs. Furthermore, the multi-target mechanism of MET against *C. albicans* ATCC 90029 is investigated mainly through changes in physiological parameters, microscopes, and transcriptome sequencing analysis. MET can inhibit biofilm formation and disrupt the mature biofilm. Simultaneously, it inhibits filamentation formation and spore germination, which is accompanied by damage to the fungi cell structure, including the cell wall, membrane, and cytoplasm. In particular, the permeability and integrity of the cell membrane are all destroyed. Once the compound MET enters the fungi cell, it disrupts redox balance by ROS accumulation and influences the energy system by activating LDH activity. More importantly, it can bind to DNA and destroy its function. Overall, MET exerts antifungal activity via a multi-target mechanism of action.

The future trends in MET-based antifungal agents may be grouped under the following topics: (I) to investigate the reasons for the observed differences in MIC values among different strains, providing potential targets and seeking to broaden the antifungal activity; (II) to explore the mechanism of action using the positive drug with clear mechanism; (III) to explore the mechanism of action by investigating enzyme inhibition activities, such as ergosterol synthetase, enzymes associated with energy metabolism, and DNA gyrase; and (IV) future structure optimization targeting DNA for this skeleton to discover more drug candidates against *C. albicans*. However, the toxicity against normal cells, tissues, and organs, as well as mutagenicity, should be considered.

## Figures and Tables

**Table 1:** The in silico prediction of toxicity.

Acute Toxicity (LD_50_)	Organ Toxicity				Mutagenicity
	Hepatotoxicity	Nephrotoxicity	Respiratory Toxicity	Cardiotoxicity	
450 mg/kg	Inactive	Inactive	Inactive	Inactive	Active

## Data Availability

Data are contained in the present work.
